# Gastric Ulcer and Cancer of the Stomach

**Published:** 1881-02

**Authors:** 


GASTRIC ULCER AND CANCER OF THE STOMACH.

In a clinical lecture on simple gastric ulcer, by Trousseau, in which the question of the diagnosis of cancer of the stomach is touched upon, the following instructive passage occurs:

"Should the cancerous tumor not be accessible to investigation, as in the case to which I have just alluded, there remains a valuable diagnostic sign which I must indicate to you. This sign, to which, over fifteen years ago, I first called the attention of the profession, consists in the appearance of a *venous thrombosis.* When you are in doubt as to the nature of a gastric disorder, and are hesitating between a chronic gastitris, a simple ulcer, and a cancer, a *phlegmasia alba dolens* of the lower or the upper extremity will put an end to your indecision, and it will be allowable for you to assert positively the existence of a cancer." Trousseau died, as is well known, of cancer of the stomach. Not long after his death, Professor Lasegue, of Paris, published an obituary notice, in which he related the following striking incident. Having described the earlier phases of Trousseau's illness, Lasegue proceeds to relate how "he was with difficulty persuaded, by the solicitations of his friends, to make a short visit a the sea-side. There a certain degree of improvement showed itself. . . . But, alas! it was only of short duration. A warning, which he less than any one could fail to understand, signified to him that thenceforth he had only to resign himself to his fate. I see him now before me, as he was on that never-to-be-forgotten day, when, taking me by the hand, he said, 'My friend, a phlebitis set in last night; it gives me but little pain; nevertheless, I know too well, by long experience, the meaning of this symptom to need any further warning.'" Trousseau's death followed not long after this interview with his friend and biographer, Lasegue.—*Boston Med. and Surg. Journ.*

